# Extracellular vesicle-derived miRNAs improve stem cell-based therapeutic approaches in muscle wasting conditions

**DOI:** 10.3389/fimmu.2022.977617

**Published:** 2022-11-14

**Authors:** Laura Yedigaryan, Ester Martínez-Sarrà, Giorgia Giacomazzi, Nefele Giarratana, Bernard K. van der Veer, Alessio Rotini, Silvia Querceto, Hanne Grosemans, Álvaro Cortés-Calabuig, Sara Salucci, Michela Battistelli, Elisabetta Falcieri, Rik Gijsbers, Mattia Quattrocelli, Kian Peng Koh, Liesbeth De Waele, Gunnar M. Buyse, Rita Derua, Maurilio Sampaolesi

**Affiliations:** ^1^ Translational Cardiomyology Laboratory, Stem Cell and Developmental Biology, Department of Development and Regeneration, KU Leuven, Leuven, Belgium; ^2^ Department of Development and Regeneration, Laboratory for Stem Cell and Developmental Epigenetics, Stem Cell Institute, KU Leuven, Leuven, Belgium; ^3^ Laboratory for Cytogenetics and Genome Research, Department of Human Genetics, KU Leuven, Leuven, Belgium; ^4^ Cellular Signalling Laboratory, Department of Biomedical and NeuroMotor Sciences, University of Bologna, Bologna, Italy; ^5^ Department of Biomolecular Sciences, Urbino University Carlo Bo, Urbino, Italy; ^6^ Laboratory for Molecular Virology and Gene Therapy, Department of Pharmaceutical and Pharmacological Sciences, Leuven Viral Vector Core, KU Leuven, Leuven, Belgium; ^7^ Cincinnati Children’s Hospital Medical Center, Department of Pediatrics, Heart Institute, University of Cincinnati College of Medicine and Molecular Cardiovascular Biology Division, Cincinnati, OH, United States; ^8^ Department of Development and Regeneration, Pediatric Neurology, University Hospitals Leuven, KU Leuven, Leuven, Belgium; ^9^ Laboratory of Protein Phosphorylation and Proteomics, Department of Cellular and Molecular Medicine, SyBioMa, KU Leuven, Leuven, Belgium; ^10^ Histology and Medical Embryology Unit, Department of Anatomy, Histology, Forensic Medicine and Orthopaedics, Sapienza University of Rome, Rome, Italy

**Keywords:** extracellular vesicle, hypertrophy, muscular dystrophy, aging, miRNA, skeletal muscle

## Abstract

Skeletal muscle holds an intrinsic capability of growth and regeneration both in physiological conditions and in case of injury. Chronic muscle illnesses, generally caused by genetic and acquired factors, lead to deconditioning of the skeletal muscle structure and function, and are associated with a significant loss in muscle mass. At the same time, progressive muscle wasting is a hallmark of aging. Given the paracrine properties of myogenic stem cells, extracellular vesicle-derived signals have been studied for their potential implication in both the pathogenesis of degenerative neuromuscular diseases and as a possible therapeutic target. In this study, we screened the content of extracellular vesicles from animal models of muscle hypertrophy and muscle wasting associated with chronic disease and aging. Analysis of the transcriptome, protein cargo, and microRNAs (miRNAs) allowed us to identify a hypertrophic miRNA signature amenable for targeting muscle wasting, consisting of miR-1 and miR-208a. We tested this signature among others *in vitro* on mesoangioblasts (MABs), vessel-associated adult stem cells, and we observed an increase in the efficiency of myogenic differentiation. Furthermore, injections of miRNA-treated MABs in aged mice resulted in an improvement in skeletal muscle features, such as muscle weight, strength, cross-sectional area, and fibrosis compared to controls. Overall, we provide evidence that the extracellular vesicle-derived miRNA signature we identified enhances the myogenic potential of myogenic stem cells.

## 1 Introduction

Muscle remodelling is a dynamic process occurring throughout the whole tissue life span. Under physiological conditions and during development and growth, skeletal muscle is finely regulated by homeostatic processes that maintain a balance between anabolic and catabolic pathways (1). During development, protein synthesis and the activation of progenitors contribute to new muscle formation. Conversely, during adulthood, cellular turnover generally decreases and muscle mass plasticity is mainly determined by the interplay between anabolism and catabolism (2). In steady state, these two pathways remain balanced. However, skeletal muscle homeostasis is disrupted when chronic damage occurs, being especially severe in degenerative diseases, such as muscular dystrophies (3). At the same time, a negative equilibrium between anabolic and catabolic factors, as well as between apoptotic and regeneration processes, is naturally associated with the progressive muscle loss that occurs during aging (4). Indeed, aging is marked by decreased function and mass across all organs. Skeletal muscle loss in aging, defined as sarcopenia, is correlated with a progressive decline in functionality and depletion of the resident stem cell pool, including the muscle-specific resident stem cells or satellite cells. Albeit still controversial, evidence has shown that a gradual decline in the number of satellite cells observed in aging is correlated with a corresponding decrease in their ability to sustain muscle regeneration upon injury (5, 6). Skeletal muscle wasting is therefore a critical condition that can be linked to degenerative diseases, be it a consequence of chronic illness such as cancer cachexia, or rather an intrinsic property of the aging process.

Concurrently, the research on the application of stem cells in muscle wasting conditions has been broad and has shown partial beneficial effects in counteracting dystrophy-induced muscle damage or other chronic muscle injuries (7). In particular, the use of mesoangioblasts (MABs), interstitial, vessel-associated adult stem cells, has given promising results when MABs were transplanted intra-arterially in murine and canine models of muscular dystrophies (8, 9). More recent evidence has shown that MABs are able to functionally fuse with resident fibers and give rise to a higher number of donor fibers when injected intramuscularly in dystrophic muscle or following acute injury (10). Additionally, the effect of aging on the potential of progenitor cells has been highlighted, as MABs derived from younger donors held greater intrinsic myogenic ability compared to MABs derived from elderly donors (11). These data strongly suggest that the potential of stem cells in contributing to muscle regeneration is decisively influenced by aging.

Considering recent evidence establishing the secretory ability of skeletal muscle, growing interest has been dedicated to the investigation of paracrine modulators, e.g. myokines, and more recently extracellular vesicles (EVs), involved in muscle regenerative processes (12, 13). Recent studies on EVs, including exosomes, have pointed out that secreted vesicles play a role in muscle physiological growth and development (14), muscle regeneration following injury or chronic illness (15, 16) and ultimately, in muscle wasting and aging (17). microRNAs (miRNAs) are among the selected cargos secreted into EVs, and their involvement in skeletal muscle homeostasis, regeneration, and wasting is rather well described (18). Interestingly, it has been reported that EVs carry members of the myomiR family of miRNAs, such as miR-1, miR-133a, and miR-206, and thus they might contribute directly to conveying regenerative signalling to the skeletal muscle (19, 20). Nonetheless, there is still a lack of consensus on the characterization of EVs (20, 21). Although studies have shown the contribution to muscle regeneration through both EVs and cell transplantation, the field is still open for further approaches, especially the use of EVs and MABs (22). Furthermore, much less evidence is available on other components of EVs’ load cargo other than miRNAs, such as mRNAs and proteins, and their possible reciprocal interaction. Finally, an interesting feature of EVs is the possibility to custom-load them for specific delivery of selected RNA or protein cargo, as shown by several studies that have paved the way for the use of EVs as a delivery machine for targeted therapeutics (23–26).

In the current study, we deeply investigated the content of plasma-derived EVs from mouse models of muscle hypertrophy, dystrophy, and age-induced atrophy in order to pinpoint the secreted key players orchestrating muscle homeostasis in physiological and pathological conditions, further amenable to enhancing muscle regeneration. We identified a hypertrophic miRNA signature capable of increasing the myogenic differentiation of human MABs (hMABs) and enhancing their myogenic contribution when transplanted *in vivo* in a murine model of acute muscle injury.

## 2 Materials and methods

### 2.1 Animal procedures

All protocols on live mice were performed in compliance with the Belgian law and the Ethical Approval of KU Leuven (P202/2016, P175/2016 with expansion). For plasma collection, 10 Myostatin-null (*Mstn-null)* (27) and 10 Met-activating genetically improved chimeric factor 1 (Magic-F1) (28) mice (hypertrophic), 10 β-sarcoglycan-null *(Sgcb-null)* (strong dystrophic phenotype) (29), 10 C57BL/6 (adult wild type (WT)), and aged 10 C57BL/6 mice were euthanized with ketamine (90 mg/kg) to preserve the rib cage, and blood was drawn by a single heart puncture. WT mice were from 3 to 6 months old, aged mice were from 13 to 20 months old, dystrophic mice were from 3 to 6 months old, and hypertrophic mice were from 3 to 6 months old. Human MABs were transfected with 30 nmol of each miRNA mimic (miR-1 and miR-208a) per well of a 12-well multiwell plate (Costar®, Corning Inc., NY, USA), with 2 µl of lipofectamine 2000 (Invitrogen, Thermo Fisher Scientific, MA, USA). *In vivo* transplantations were conducted 48 hours after transfection. For cell transplantation, aged *Rag2-null*/*ᵞc-null*/C57BL/6 mice (≥ 18 months old) received intramuscular (tibialis anterior (TA), gastrocnemius (GM), and quadriceps (Q)) cardiotoxin (10 μM) injections 48 hours prior to cell transplantation and at the day of cell transplantation, the animals received intramuscular injections of 2.5 x 10^5^ miRNA-treated MABs or intramuscular injections of 2.5 x 10^5^ non-treated MABs (n=5 per group). Muscles injected with physiological solution were used as control (cardiotoxin control). At days 21 and 35, mice were euthanized by cervical dislocation and hindlimb muscles were harvested and individually weighed. The extensor digitorum longus (EDL) muscle was immediately processed for functional analysis (Aurora, Shanghai, China). TA and GM muscles were included in OCT (Tissue-Tek® O.C.T. Compound, Sakura Finetek, Tokyo, Japan) and kept at -80°C until further analysis. The samples were then cut transversally in 7 µm sections using a cryostat machine (Leica, Wetzlar, Germany).

### 2.2 EV isolation and characterization

EVs were extracted from mouse plasma (1 - 1.5 mL per mouse and 2-3 mice per sample) of age-matched control C57BL/6 mice, *Mstn-null* mice, Magic-F1 mice, *Sgcb-null* mice, and aged C57BL/6 mice following established protocols of ultracentrifugation (30). Briefly, plasma was centrifuged for 5 minutes at 480g (Eppendorf Centrifuge, Model 5810, Eppendorf), 10 minutes at 2,000g (Avanti® J-E high-speed centrifuge, Beckman Coulter Life Sciences, IN, USA) (JA-20 fixed-angle rotor), 30 minutes at 10,000g (Beckman Coulter Life Sciences), and lastly, the supernatants were centrifuged at 100,000g for 1 hour (Optima L-90K Ultracentrifuge, Beckman Coulter Life Sciences) (70Ti fixed-angle rotor). All centrifugations were conducted at 4°C. EV pellets were resuspended in phosphate-buffered saline (PBS) (Gibco, Thermo Fisher) and stored at -80°C until further use. Transmission electron microscopy (TEM) images were obtained, as previously described (31). Nanoparticle tracking analysis was performed with the NanoSight LM10 from NanoSight Ltd. (Malvern Panalytical, Malvern, UK), software version 2.3, in collaboration with the Laboratory of Lipid Metabolism and Cancer, KU Leuven. EVs resuspended in PBS were diluted accordingly and applied to the machine for particle concentration and size determination.

### 2.3 Cell culture and differentiation

Human MABs were isolated as previously described (32). MABs were cultured in IMDM supplemented with 15% fetal bovine serum (FBS), 1% penicillin/streptomycin (Pen/Strep), 1% L-glutamine, 1% sodium pyruvate, 1% non-essential amino acids, 1% insulin-transferrin-selenium, and 0.2% b-mercaptoethanol (all reagents from Life Technologies, Thermo Fisher) (Gibco) in 5% O_2_/5% CO_2_ at 37°C. Mouse myoblast cell line C2C12 was cultured in DMEM supplemented with 10% FBS, 1% Pen/Strep, 1% L-glutamine, 1% sodium pyruvate, 1% non-essential amino acids, and 0.2% b-mercaptoethanol (all reagents from Life Technologies) in 5% O_2_/5% CO_2_ at 37°C. Myogenic differentiation of MABs and C2C12s was induced by incubating the cells with DMEM high glucose, supplemented with 2% of Horse Serum, 1% Pen/Strep, 2 mM L-glutamine, and 1 mM sodium pyruvate (all reagents from Life Technologies) for 5 days (C2C12 cells) and 12 days (MABs).

### 2.4 EV treatment

The ExoGlow™ Red reagent was utilized to label EVs following manufacturers’ instructions (System Biosciences, CA, USA). C2C12 myoblasts were treated for 4 hours with red fluorescent protein (RFP)-marked EVs (1x10^8^) and RFP signal in the cells’ cytoplasm was detected and pictures were taken with a Nikon Eclipse Ti microscope (Nikon, Tokyo, Japan) and a Leica TCS SP8 X confocal microscope (Leica). All cells were positive for RFP-EVs (most pictures are 100%). However, some cells showed more concentration than others, therefore, the quantification was done in terms of % of RFP-positive area divided by the area covered by all cells per field of view (n = 4). C2C12s and MABs were exposed for 48 hours to EVs (1x10^8^) before inducing myogenic differentiation.

### 2.5 Molecular analysis

Western blotting (WB) analysis was performed on lysates from EVs (1x10^5^) and cells in RIPA buffer (Sigma-Aldrich, MI, USA) supplemented with 10 mM sodium fluoride, 0.5 mM sodium orthovanadate, 1:100 Halt™ Protease Inhibitor Cocktail, and 1 mM phenylmethylsulfonyl fluoride (all reagents from Thermo Fisher Scientific). Equal amounts of protein (30 μg) (Bradford assay, Quick Start™ Bradford 1x Dye Reagent, Bio-Rad Laboratories, Inc. CA, USA) were denaturized in sample-loading buffer (50 mM Tris–HCl, pH 6.8, 100 mM dithiothreitol (DTT), 2% SDS, 0.1% bromophenol blue, 10% glycerol (all reagents from Sigma-Aldrich) for 10 minutes at 95°C), run on SDS-polyacrylamide gels, and transferred to nitrocellulose membranes (Amersham™ Protran® 0,45 μm NC, GE Healthcare Lifesciences, NJ, USA). After Tris-buffered saline (TBS) containing 0.05% Tween and 5% non-fat dry milk (Sigma-Aldrich) background blocking, membranes were incubated overnight with primary antibody (CD9, CD63, CD81, GM130, HSP90, Calnexin (Invitrogen), myosin heavy chain (MyHC) (in-house hybridoma), α-Tubulin (Cell Signaling Technology, MA, USA), and GAPDH (Sigma-Aldrich)). Following secondary antibody incubation (anti-mouse (1:5000, Goat Anti-Mouse IgG (H + L)-HRP Conjugate #1706516, Bio-Rad Laboratories, Inc., Hercules) and anti-rabbit (1:5000, Goat Anti-Rabbit IgG (H + L)-HRP Conjugate #1706515, Bio-Rad Laboratories, Inc.)) in TBS-Tween and 2.5% non-fat dry milk, membranes were incubated with SuperSignal Femto or Pico (Thermo Scientific) and bands were detected with a GelDoc chemiluminescence detection system (Bio-Rad Laboratories, Inc.).

miRNAs were isolated from EVs by using the mirVana™ miRNA isolation kit (Invitrogen). cDNA synthesis was performed using the TaqMan™ Advanced miRNA cDNA Synthesis Kit (Applied Biosystems, Thermo Fisher) and qPCR was performed using TaqMan™ Advanced miRNA Assays (Applied Biosystems) using 1:15 diluted cDNA obtained from 20 ng total of miRNA preparation (quantified by the Nanodrop™ 1000 (Thermo Scientific). miRNA content in EVs was analyzed by the mmu-miRNome micro-RNA profiling kit (Systems Biosciences) following the manufacturers’ instructions (n=4).

### 2.6 Immunofluorescence, immunohistochemistry, and microscopy

Cells for *in vitro* analyses and slides for *in vivo* analyses were fixed with 4% paraformaldehyde (Sigma-Aldrich) for 15 minutes at room temperature, washed with PBS, permeabilized with 1% bovine serum albumin (BSA) in PBS + 0.2-0.5% Triton™ X-100 (Sigma-Aldrich) for 30-45 minutes at room temperature, background blocking was performed for 30 minutes with 10% donkey serum (Biowest, Nuaillé, France) solution at room temperature and samples were incubated overnight at 4°C with the primary antibody (MyHC (1:20, in-house hybridoma), human lamin A/C (hLMNA) (1:200, NCL-LAM-A/C, Novocastra Laboratories Ltd, Newcastle, UK), laminin (1:300, L9393, Sigma-Aldrich)). The following day, after PBS washes, samples were incubated for 1 hour at room temperature with the Alexa Fluor™-conjugated donkey secondary antibody (1:500, Invitrogen) and washed. Hoechst 33342 Solution (20 mM) (Hoechst) (1:3000, #62249, Thermo Scientific) was used, washed, and the immunofluorescence was mounted using FluorSave™ reagent (Merck Millipore, MA, USA).

Pictures were acquired on a Nikon Eclipse Ti microscope (Nikon). Pictures of hMABs *in vitro* differentiation assays were acquired on a Nikon Ti2 automated fluorescent image scanner, pre-specified at 4 x 4 fields per well, centered on a 24-well format using an automated x-y motorized stage. Afterwards, images acquired per well were electronically stitched using NIS-Elements software (Nikon) for full-well image reconstitution.

Haematoxylin and eosin staining was performed as previously described (33). Briefly, cryosections were fixed with 4% paraformaldehyde for 15 minutes. Slides were then soaked in distilled water for 5 minutes, then Harris haematoxylin for 4 minutes, and washed afterwards in running water for 2 minutes. Afterwards, the sections were immersed for 1 minute each in acid alcohol, running water, bluing reagent, running water, eosin, 95% ethanol, 100% ethanol, and histoclear, and were finally mounted with dibutylphthalate polystyrene xylene (DPX) and left on a slide heater overnight. All reagents were purchased from Sigma-Aldrich. Pictures from cryosections were taken with a Nikon Eclipse Ti microscope (Nikon) and a Zeiss Axio Imager Z1 (Zeiss, Jena, Germany). The cross-sectional area was determined using ImageJ software (NIH, MD, USA).

Masson’s trichrome was performed as previously reported (34). Briefly, after 15 minutes in 4% paraformaldehyde of fixing, cryosections were incubated for 15 min at 57°C in Bouin’s Solution and subsequently stained in Working Weigert’s Iron Hematoxylin Solution for 5 minutes, washed in running tap water for 5 minutes, and stained in Biebrich Scarlet-Acid Fuchsin for 5 minutes. Afterwards, slides were soaked in de-ionized water and in Working Phosphotungstic\Phosphomolybdic Acid Solution for 5 minutes and stained in Aniline Blue Solution for 5 minutes and 1% acid acetic for 2 minutes. Slides were mounted with DPX. All reagents were purchased from Sigma-Aldrich. After mounting, pictures were taken on a Nikon Eclipse Ti microscope (Nikon) and a Zeiss Axio Imager Z1 (Zeiss). The fibrotic area was quantified using ImageJ software (NIH).

### 2.7 RNA sequencing (RNA-seq)

Total RNA and proteins were isolated from EVs using the Total Exosome RNA & Protein Isolation Kit (Invitrogen) and following manufacturers’ instructions. Samples were verified and processed by the Genomics Core (KU Leuven, UZ Leuven, Belgium). A preamplification step was required before sequencing due to low concentrations of RNA (< 5µg/µl). RNA-seq libraries were constructed with the TruSeq RNA Sample Prep Kits v2 (Illumina, CA, USA). Samples were indexed with unique adapters and pooled for single-read (50 bp) sequencing in an Illumina HiSeq 2000 sequencing system (Illumina). RNA-seq reads were aligned with TopHat v2.0.2 to the mouse genome version mm10. Differential expression levels were assessed using DESeq (n=6 *Mstn-null*-derived samples, n=6 *Sgcb-null*-derived, and n=3 wild type-derived). After no identification of differentially expressed genes, we further selected a cut-off of ≥ 50 reads and performed GO analysis by means of BinGO. The data discussed in this publication have been deposited in NCBI's Gene Expression Omnibus (35) and are accessible through GEO Series accession number GSE216414 (https://www.ncbi.nlm.nih.gov/geo/query/acc.cgi?acc=GSE216414).

### 2.8 EV mass spectrometry analysis

EVs (5x10^9^) were lysed in a buffer containing 7M urea, 50 mM Tris-HCl pH 7.4, 150 mM NaCl, 15 mM EDTA, 1% NP-40 (Sigma-Aldrich), and a mixture of protease inhibitors (cOmplete™ protease inhibitor, Roche Diagnostics, Basel, Switzerland). Lysates were reduced, alkylated, protein precipitated, and trypsin digested and the resulting peptides were subjected to C18 (Pierce™ C18 Spin Tips & Columns, Thermo Fisher) cleanup as previously described (36). Briefly, lysates were reduced in 5 mmol/L DTT for 30 min at 37°C followed by alkylation with 25 mmol/L iodoacetamide (IAA) (Sigma-Aldrich) and quenching of excess IAA with 25 mmol/L DTT, both by incubating for 30 min at 37°C in the dark. Protein precipitation was performed using the Wessel-Flügge method (37). Digestion was performed with modified trypsin (Pierce™ Trypsin Protease, MS Grade, Thermo Fisher) in 200 mmol/L ammonium bicarbonate (pH 8) in the presence of 5% acetonitrile (ACN) and 0.01% ProteaseMAX™ Surfactant, Trypsin Enhancer (Promega, WI, USA). The solvent-evaporated peptide mixtures were subjected to desalting with C18 ZipTip® pipette tips (Merck Millipore).

The resulting peptide mixture was separated on an Ultimate 3000 UHPLC system (Dionex, Thermo Scientific) equipped with an Acclaim™ PepMap100™ pre-column (C18, particle size 3 μm, pore size 100 Å, diameter 75 μm, length 20 mm, Thermo Scientific) and a C18 PepMap™ analytical column (particle size 2 μm, pore size 100 Å, diameter 50 μm, length 150 mm, Thermo Scientific) using a 40 min linear gradient (300 nL/min) before being analyzed by a Q Exactive Orbitrap mass spectrometer (Thermo Scientific) in data-dependent acquisition mode.

Peptides were identified by Mascot (Matrix Science) using Uniprot *Mus musculus* concatenated with the Crapome contaminants database (115 142 sequences) as a database. Carbamidomethylation (C) was included as a fixed modification and Oxidation (M) as a variable modification. Two missed cleavages were allowed and peptide tolerance was set at 10 ppm and 20 mmu for MS and MS/MS tolerance, respectively.

Relative quantification of proteins was executed using Scaffold_4.11.1 software (Proteome Software, Inc., OR, USA). Only proteins (no crapome hits) containing at least 2 identified peptides with a false discovery rate (FDR) < 1% and displaying 95% protein probability (or more) were taken into consideration. For drawing the Venn diagram, only proteins showing spectral counts > 0 in at least two of the three replicates, were taken into account. Qlucore software (Qlucore, Lund, Sweden) was used for statistical data analysis using Scaffold ‘normalized total spectra’ as input. The Venn diagram was drawn using http://bioinformatics.psb.ugent.be/webtools/Venn/. The mass spectrometry proteomics data have been deposited to the ProteomeXchange Consortium *via* the PRIDE (38) partner repository with the dataset identifier PXD035446 and 10.6019/PXD035446.

### 2.9 MicroRNA modulation

Human MABs were transfected with 30 nmol of each miRNA mimic (miR-1, miR-208a, miR-133a, and/or miR-206) (MISSION® microRNA Mimic, Sigma-Aldrich) (HMI0057, HMI0365, HMI0196, and HMI0364) and 10 nmol of each antagomir (miR-1, miR-208a, miR-133a, and/or miR-206) (MISSION® Synthetic microRNA Inhibitor, Human, Sigma-Aldrich) (HSTUD0046, HSTUD0365, HSTUD0196, and HSTUD0364) per well of a 12-well multiwell plate (Costar®, Corning Inc.), with 2 µl of lipofectamine 2000 (Invitrogen). miRNA expression analysis, differentiation assays, and *in vivo* transplantation were conducted 48 hours after transfection.

### 2.10 Viral vector transduction

To enable tracking of hMABs after injection *in vivo* and in real-time and to prove cell viability, hMABs were transduced with a murine leukemia virus (MLV)-derived retroviral vector containing enhanced green fluorescent protein (eGFP)-T2A-firefly luciferase (fLuc) (constructed and produced by the Leuven Viral Vector Core) applied at a 1:200 concentration for 72 hours (2.34e+08 TU/ml).

### 2.11 Distribution and viability of eGFP/fLuc human MABs *in vivo*

For *in vivo* bioluminescence imaging (BLI), mice were first anesthetized with 1.5% of isoflurane (Iso-Vet®, 100 mg/ml, Piramal Healthcare, London, UK) in 100% oxygen (flow rate of 2 L/min) and then given a single intravenous injection containing d-luciferin potassium salt (Promega, WI, USA) dissolved in PBS (126 mg/kg, 10 µL/g). Ten minutes after luciferin injection, mice were placed in the imaging chamber (IVIS® Spectrum, Perkin-Elmer, MA, USA). Next, consecutive frames were acquired for 60 seconds until the maximum signal intensity was reached. After drawing a region of interest (ROI) around the hindlimb muscles, the maximal radiance (p/s/cm^2^/sr) was measured within this region, and subsequent images were analyzed using Living Image® version 4.5 (Caliper Life Sciences, MA, USA). BLI data were obtained 1h, 1,3,7,14, and 21 days post injections. Mice were euthanized by cervical dislocation immediately after *in vivo* BLI and the hindlimb muscles were collected in OCT (Sakura Finetek).

### 2.12 Muscle function by an intact muscle test system (Aurora Scientific Instruments, Aurora, Canada)

EDL functionality was evaluated as previously described (39). Briefly, the EDL was immediately excised from each mouse, maintained in a storage solution in a temperature-controlled (30 °C) chamber containing the buffer solution, and continuously gassed with a mixture of 95% O_2_ and 5% CO_2._ The muscle was initially held for isometric contraction and was stimulated with a 0.5 ms single pulse to measure the isometric twitch force and contraction time. A second pulse was applied to check the consistency of the values obtained and to better balance muscle equilibration before applying tetanic stimulation.

The muscle was then subjected to a first train (0.6 s at 120 Hz) to induce unfused tetanus and to a second train (0.6 s at 180 Hz) to evoke the maximal tetanic force.

### 2.13 Statistical analysis

Statistical analysis and graphing of the results were performed using GraphPad Prism 7.0 (GraphPad Software, CA, USA). Two-tailed *t*-test or one-way ANOVA was used to compare interrelated samples, while two-way ANOVA was used to analyze multiple factors. Confidence intervals were fixed at 95% (*p* < 0.05). Data are reported as mean ± standard error of the mean (s.e.m). Refer to figure legends for specific information regarding the statistical test used.

## 3 Results

### 3.1 Characterization of extracellular vesicles from wild type, hypertrophic, dystrophic, and aged mice

**Figure 1:**
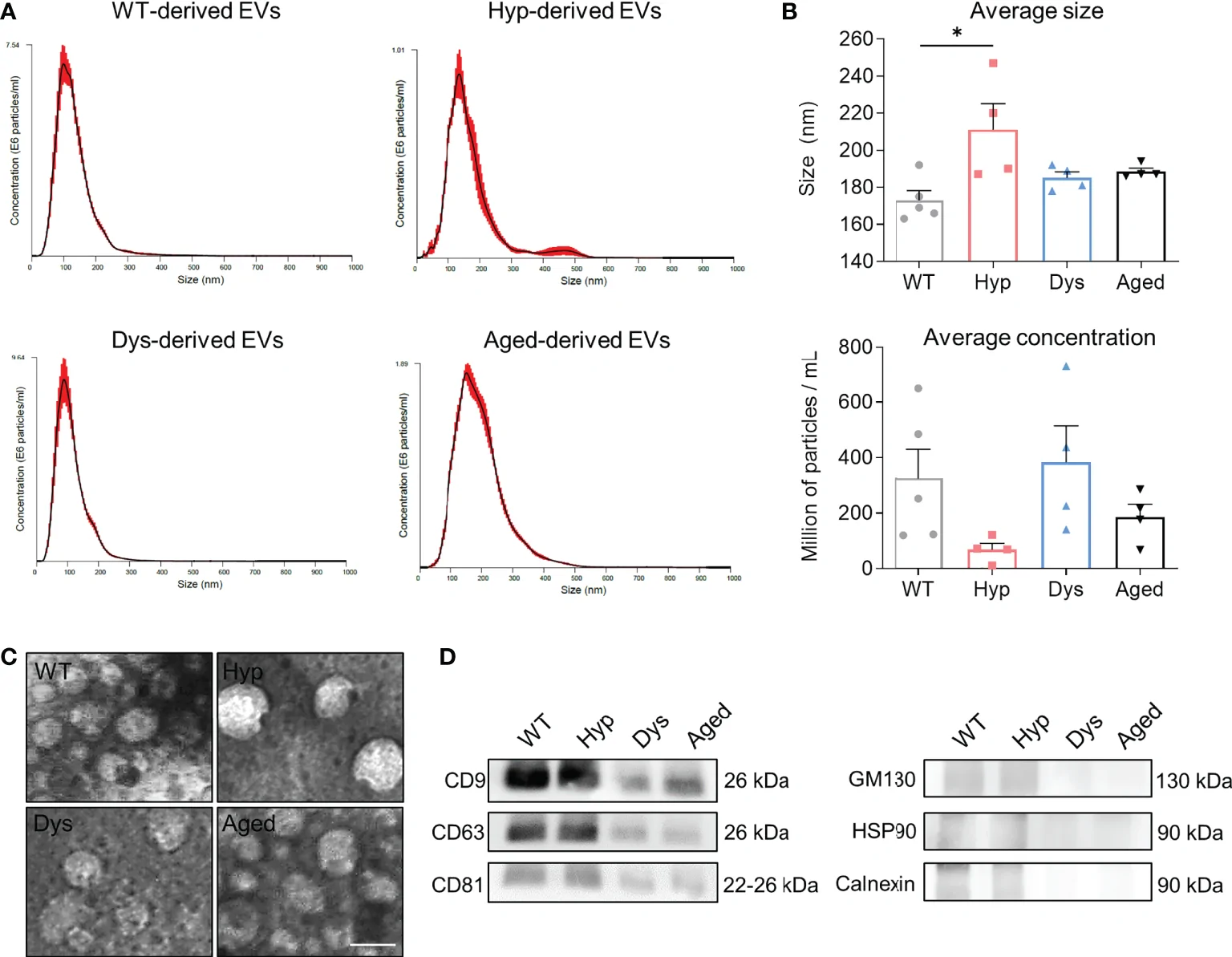
Characterization of extracellular vesicles derived from the plasma of different mouse models. **(A)** NanoSight analysis of the obtained extracellular vesicles (EVs), representative graphs of particle sizes in wild type (WT)-, hypertrophic (Hyp)-, dystrophic (Dys)-, and aged-derived EVs are shown. **(B)** Average size (upper graph) and concentration (lower graph) of EVs. One-way ANOVA was used and results are displayed as mean ± s.e.m (n=4 samples/group, EVs from 2-3 mice/sample; *p < 0.05). **(C)** Transmission electron microscopy analysis of EVs. Scale bars: 100 nm. **(D)** Western blot analysis of common positive (CD9, CD63, CD81) and negative (GM130, HSP90, Calnexin) markers for EVs and/or exosomes.

In order to characterize the EV content from different mouse models, we started our analysis by isolating EVs from the plasma of age-matched control C57/bl6 mice (wild type; WT), *Mstn-null* mice (hypertrophic (Hyp)), *Sgcb-null* mice (dystrophic (Dys)), and aged C57/bl6 mice. EVs were characterized by NanoSight analysis, which revealed an average size of 100-200 nm for WT, dystrophic, and aged-derived EVs, further confirmed by electron microscopy analysis (**Figures 1A–C**). We observed that hypertrophic-derived EVs seem to be lower in concentration and this correlated with a significantly higher average size (**Figures 1B, C**). We further characterized these EVs for known extracellular vesicle and exosome markers, and all samples were positive for CD81, CD9, CD63, and negative for Calnexin, HSP90, and GM130 (**Figure 1D**).

### 3.2 Extracellular vesicles from hypertrophic mice enhance myogenic differentiation in myogenic progenitors *in vitro*

**Figure 2:**
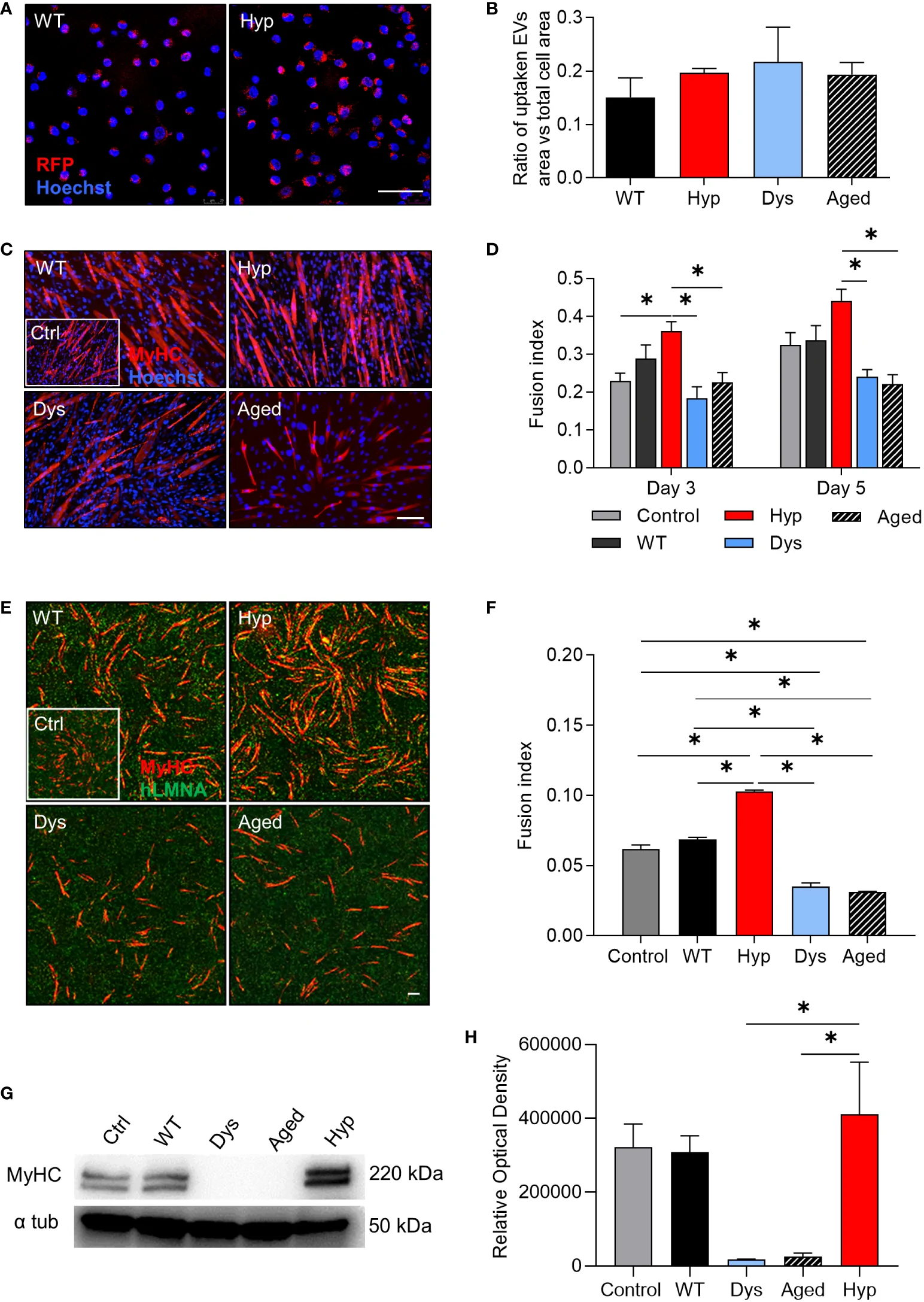
Effect of extracellular vesicle treatment on myogenic differentiation of progenitor cells. **(A)** Red fluorescent protein-marked extracellular vesicle (EV) uptake (red) from mouse myoblast cell line C2C12 after 4 hours of EV treatment. Representative confocal images of cells treated with wild type (WT)- and hypertrophic (Hyp)-derived EV samples are shown. Hoechst was counterstained in blue. **(B)** Quantification of EV uptake in C2C12 myoblasts after 4 hours of EV treatment (n=4 per group). **(C)** Myogenic differentiation of C2C12 cells incubated with WT-, Hyp-, dystrophic (Dys)-, and aged-derived EVs. Immunofluorescence analysis for myosin heavy chain (MyHC) (red). Hoechst was counterstained in blue. **(D)** Fusion index of differentiated C2C12 cells at days 3 and 5 of myogenic differentiation (n = 5). **(E)** Myogenic differentiation of human mesoangioblasts (hMABs) treated with EVs. Immunofluorescence analysis for MyHC (red) and human lamin A/C (green). **(F)** Fusion index of differentiated hMABs at day 12 (n = 3). **(G)** Example of a western blot for MyHC protein level at day 12 of myogenic differentiation. GAPDH was used as a loading control. **(H)** Quantification of MyHC western blot. For a, c, and e, scale bars: 50 µm. For b, d, f, and h one-way ANOVA was used and results are displayed as mean ± s.e.m (n=3-5, *p < 0.05).

We next tested the functional effect of EVs on murine myoblasts (C2C12 cells) and hMABs (**Figure 2**). First, we confirmed the ability of the myoblasts to uptake RFP-marked EVs to nearly 100% of efficiency in all conditions. Indeed, after 4 hours of EV treatment, we could successfully detect EVs in the cytoplasm of the treated cells (**Figures 2A, B**) and no differences were observed in terms of RPF-positive area vs total cell area across all samples (**Figure 2B**). After 48 hours of EV exposure, we induced myogenic differentiation in C2C12 cells. Our results showed that C2C12 cells treated with hypertrophic-derived EVs differentiated more efficiently, as reported by the increase in fusion index (the number of nuclei inside MyHC-positive myotubes (if 2 or more) over the total number of nuclei in a field of view) at day 3 and 5 of differentiation (**Figures 2C, D**). In contrast, cells treated with dystrophic and aged-derived EVs showed a decrease in fusion index at day 5 of differentiation (**Figures 2C, D**). In order to confirm our observation in murine myoblasts, we next tested the effects of EVs on hMABs (**Figures 2E, F**). In accordance with our previous findings, hMABs treated with hypertrophic-derived EVs 48 hours before differentiation displayed an increased myogenic differentiation as shown by immunofluorescence for MyHC and fusion index analyses at day 5 of differentiation (**Figures 2E, F**). Additionally, the exposure to dystrophic- and aged-derived EVs seemed to result in a detrimental effect on hMABs myogenic differentiation, when compared to controls and cells treated with hypertrophic-derived EVs (**Figures 2E, F**). MyHC expression was also altered upon pre-treatment with EVs (**Figure 2G**). Levels of MyHC protein were significantly higher in hypertrophic-derived EVs compared to dystrophic- and aged-derived EVs (**Figure 2H**). Our results show that hypertrophic-derived EVs modulate the differentiation of myogenic progenitors *in vitro*.

### 3.3 RNAs are randomly integrated in circulating EVs

After observing the effects of hypertrophic-derived EVs, we next wondered which factors might be responsible for the observed phenotype. Hence, we deeply analyzed the content of the EVs in terms of mRNAs, proteins, and miRNAs. Bulk RNA-seq revealed that the mRNAs loaded into EVs from the different conditions were not discriminative of each condition, suggesting that mRNAs are not likely driving the enhanced differentiation observed in C2C12s and hMABs upon EV treatment. Our results showed that EVs from all conditions were rather low in content of mRNA, with about 2000 to 6000 uniquely aligned transcripts. Interestingly, hypertrophic-derived EVs contained more than double the number of mRNAs than control WT-derived and dystrophic-derived EVs (**Supplementary Figure 1A**). Principal component analysis revealed a rather contingent clustering of the samples, suggesting that the mRNA profile was very variable within each condition (**Supplementary Figure 1B**). Based on this observation, a differentially expressed analysis of the transcripts would not lead to any solid conclusions, therefore we opted for deeply profiling the content of each condition. Analysis of the 30 most abundant transcripts in each condition revealed that all samples expressed medium to high levels of mitochondrial RNA and hemoglobin Hbb-bs (**Supplementary Figure 1D**). In order to understand better the specific content of our samples, we selected a cut-off of >50 reads and analyzed the expressed genes. Notably, only 2.3% of the genes were shared between all conditions, and while dystrophic and WT-derived EVs shared about 25% of genes, hypertrophic-derived EVs only had 1.3% and 3.9% of genes in common with WT and dystrophic-derived EVs, respectively (**Supplementary Figure 1C**). Our analysis suggests that the incorporation of mRNA in the EVs cargo is a stochastic event that might depend on multiple factors and might not be necessarily related to the underlying phenotype.

### 3.4 Characterization of the protein content of EVs

To identify EV-specific protein signatures, mass spectrometry analysis was performed. Mass spectrometry analysis on EVs from all conditions allowed the identification of 154 protein clusters in total, only considering proteins (no crapome hits) containing at least 2 identified peptides with an FDR < 1% and displaying 95% protein probability (or more). Using a cut-off value based on spectral counts (spectral counts > 0 in at least two replicates per condition), 133 protein clusters were withheld of which 79 were present in all conditions, 3 were specific for Hyp-derived EVs, 9 for WT-derived EVs, 2 were only found in EVs from aged mice, and 8 in Dys-derived EVs (**Supplementary Figure 1E**). Surprisingly, we detected a common marker in all preparations, CD5 antigen-like, as well as a high number of immunoglobulins (23%, 28%, 30%, and 34% of all protein clusters in Hyp, Dys, Aged, and WT, respectively) (data not shown). Immunoprecipitation of EVs revealed that immunoglobulins were not present on the surface of the EVs (data not shown). Applying a Kruskal-Wallis non-parametric analysis on the total list of identified proteins, 2 of them showed a significant (p < 0.05) difference among the different conditions: integrin alpha 2b and antithrombin-III (**Supplementary Figure 1F**). Integrin alpha 2b showed increased levels in EVs of dystrophic mice and, to a lesser extent, in hypertrophic samples, but was completely absent in EVs of aged and WT mice. Antithrombin-III showed increased levels in hypertrophic mice.

However, none of these proteins had any significant relevance to muscle-related conditions. Therefore, it is unlikely that the EV-derived proteins caused the hypertrophic phenotype we observed *in vitro*.

### 3.5 miRNAs derived from EVs influence the myogenic propensity of target cells

**Figure 3:**
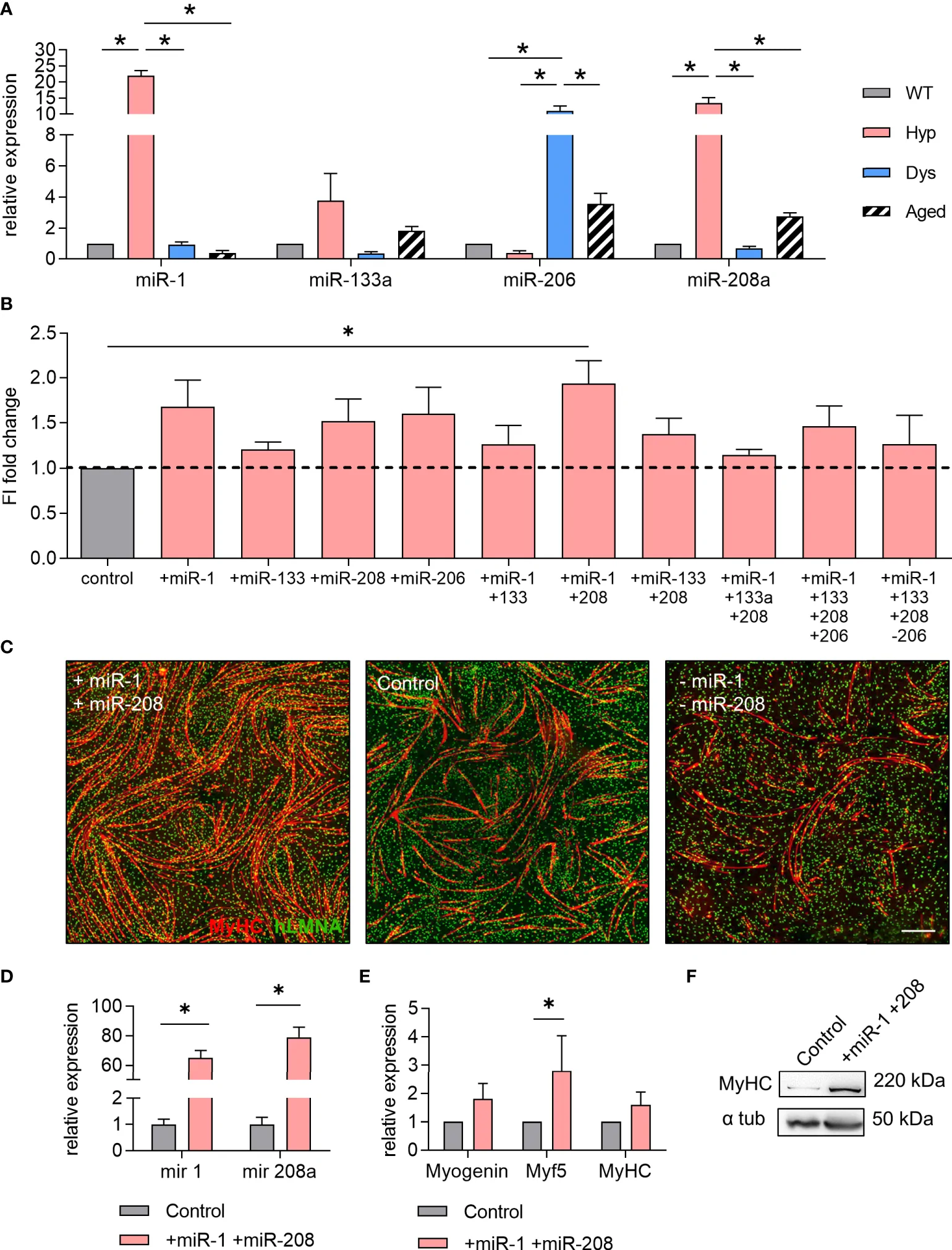
miR-1 and miR-208a increase the myogenic differentiation of human mesoangioblasts *in vitro.* **(A)** TaqMan qPCR analysis for the quantification of the selected microRNAs (miRNAs) miR-1, miR-133a, miR-206, and miR-208a in wild type (WT)-, hypertrophic (Hyp)-, dystrophic (Dys)-, and aged-derived extracellular vesicles (EVs). One-way ANOVA was used and results are displayed as mean ± s.e.m (n = 5, *p < 0.05). **(B)** Fusion index of differentiated human mesoangioblasts (hMABs) treated with combinations of miRNA mimics and antagomirs for miR-1, miR-133a, miR-206, and miR-208a (mimics are denoted by a + sign, antagomirs by a – sign). One-way ANOVA was used and results are displayed as mean ± s.e.m (n = 4, *p < 0.05). **(C)** Representative images of immunofluorescence analysis for myosin heavy chain (MyHC) (red) and human lamin A/C (green) in differentiated hMABs after miRNA treatments with miRNA mimics and antagomirs for miR-1 and miR-208. Hoechst was counterstained in blue. Scale bars: 100 µm. **(D–F)** Differentiation of hMABs treated with miR-1 and miR-208 mimics resulted in higher levels of miR-1 and miR-208 **(D)** and an increase in mRNA expression **(E)** and protein level **(F)** of myogenic markers. Two-tailed Student’s t-test was used and results are displayed as mean ± s.e.m (n = 5, *p < 0.05).

Since miRNAs have been heavily implicated in orchestrating myogenesis, we focused on the potential involvement of muscle-specific miRNAs in the previously observed phenomena (**Figure 2**). We began our analysis by investigating miR-208a, miR-1, miR-133a, and miR-206, all of them described to be implicated in myogenesis and myogenic differentiation. We next validated the expression levels of these miRNAs by means of TaqMan-based qPCR, and we confirmed high levels of miR-1, miR-133a, and miR-208a in hypertrophic-derived EVs compared to the other conditions, while high levels of miR-206 could be found in dystrophic-derived EVs compared to the other conditions (**Figure 3A**). Furthermore, we checked the expression of such miRNAs in skeletal muscle tissue to elucidate whether the presence of the determined miRNAs in the plasma-derived EVs could be tissue-specific (**Supplementary Figure 3A**). These results suggest that the content of miRNAs loaded into the EVs is directly correlated to the content of muscular miRNAs, and further suggest that the origin of the EVs enriched with the selected miRNAs could be the skeletal muscle. Interestingly, when analyzing the miRNA content of plasma-derived EVs from a different mouse model of muscle hypertrophy (Magic-F1) (40), we identified the very same miRNA signature, namely an upregulation of miR-1 and miR-208a and downregulation of miR-206 compared to control WT-derived EVs (**Supplementary Figure 3B**).

We next tested the effects of this set of miRNAs on hMABs: combining mimics and antagomir oligonucleotides, we aimed to upregulate and downregulate miR-1, miR-133a, miR-206, and miR-208a using several different combinations of them (**Figure 3B**). Our results showed that from all the different combinations tested, the simultaneous upregulation of miR-1 and miR-208a significantly increased the myogenic differentiation of hMABs resulting in a 2-fold increase of the fusion index at day 12 of differentiation (**Figures 3B, C**). Upregulation of miR-1 and miR-208a was confirmed (**Figure 3D**) and resulted in a significant increase in Myf5 gene expression (**Figure 3E**). Furthermore, protein detection by WB analysis in differentiated hMABs treated with miR-1 and miR-208a compared to non-treated hMABs additionally confirmed these results (**Figure 3F**). Taken together, our results indicate that the enhanced myogenic differentiation of myogenic progenitors observed *in vitro* upon treatment with EVs is driven by miRNAs, with miR-1 and miR-208a being the main candidates.

In order to get a better understanding of miRNAs included in the EVs from all conditions, we deeply profiled the miRNA content by performing a SYBR green I-based analysis of >700 miRNAs present in EVs (**Supplementary Figure 2A**). Our high-throughput analysis did not show clear differences among samples; however, a small set of miRNAs were found more abundant in the circulating EVs from *Mstn-null* mice. In particular, miR-150* and miR-30e* were highly enriched in hypertrophic samples, while miR-574-5p was downregulated in hypertrophic samples (**Supplementary Figure 2B**). However, upon modulation of these miRNAs on the *in vitro* differentiation of MABs, we did not detect any myogenic effects (**Supplementary Figures 2C, D**).

### 3.6 EV-derived microRNA modulation of human mesoangioblasts is beneficial for muscle regeneration *in vivo* in cardiotoxin-injured aged mice

**Figure 4:**
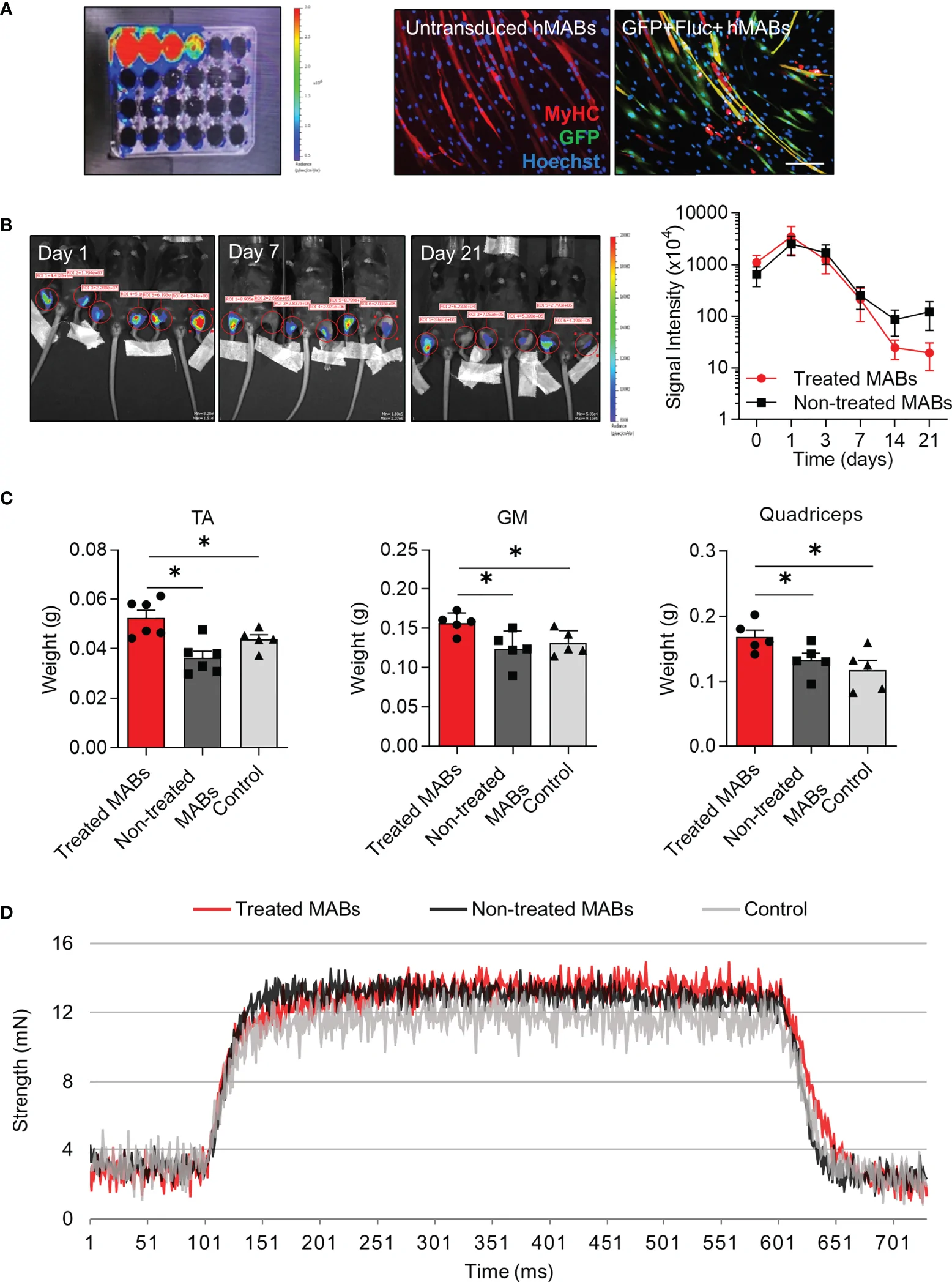
Cell transplantation of microRNA-treated and non-treated human mesoangioblasts in aged mice after acute injury. **(A)** Bioluminescence of human mesoangioblasts (hMABs) cultured *in vitro* transduced with the enhanced green fluorescent protein (eGFP)/firefly luciferase vector (left) and immunofluorescence analysis for myosin heavy chain (MyHC) (red) and GFP (green) in differentiated hMABs with and without transduction. Hoechst was counterstained in blue. Scale bars: 50 µm. **(B)** *In vivo* bioluminescence imaging at days 7, 14, and 21 in injected mice (left) and signal quantification (right). Left limbs received microRNA-treated MABs, right limbs received non-treated MABs. **(C)** Weight of hindlimb muscles after mice were sacrificed at day 21. TA: Tibialis anterior, GM: Gastrocnemius. **(D)** Functional analysis of extensor digitorum longus *via* evaluation of tetanic force. One-way ANOVA was used and results are displayed as mean ± s.e.m (n = 5-6 muscles/group, *p < 0.05).

**Figure 5:**
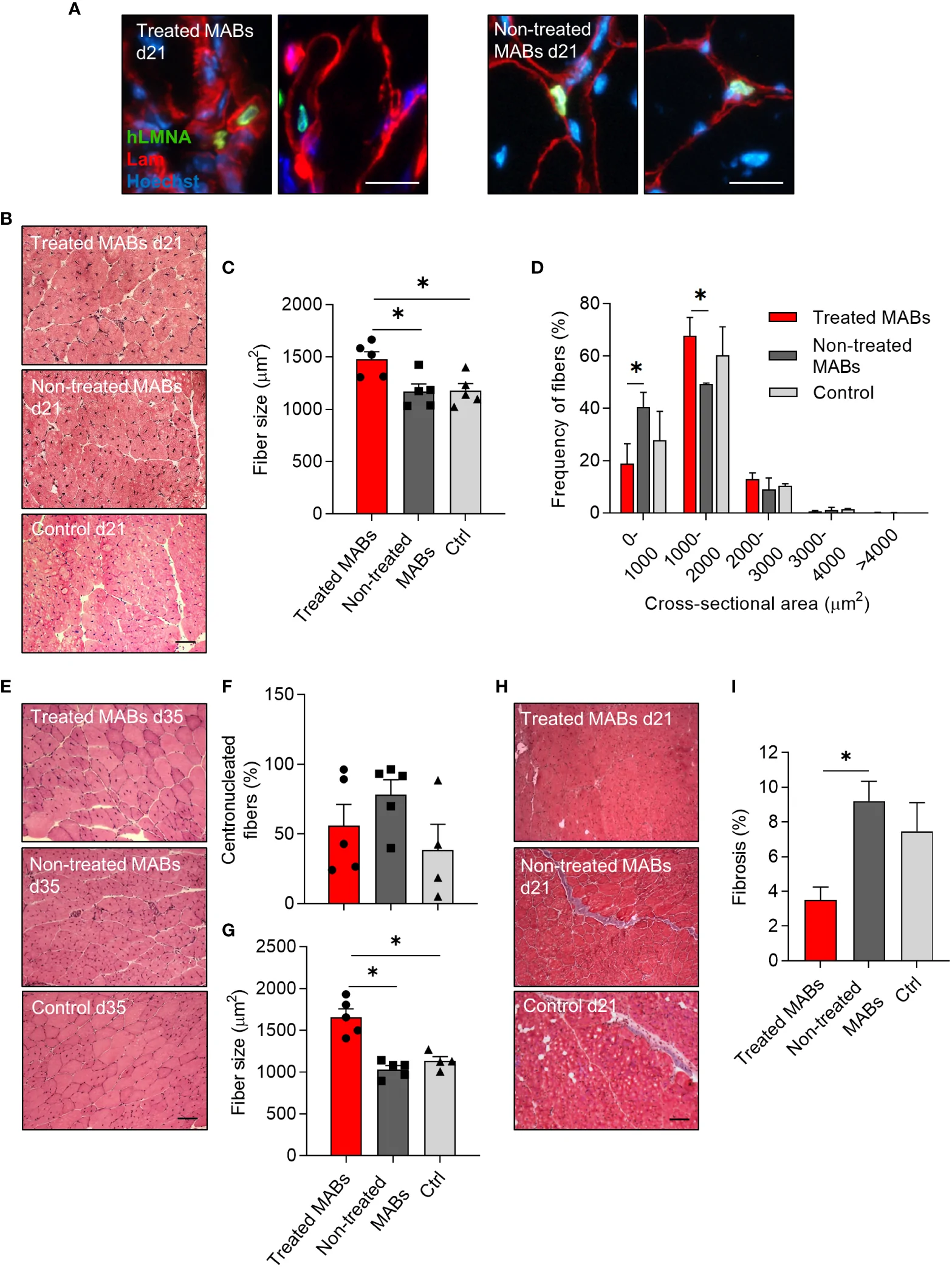
Histological analysis of aged muscles after acute injury and cell transplantation of microRNA-treated and non-treated human mesoangioblasts. **(A)** Localization of human mesoangioblasts (hMABs) in the injected skeletal muscle at day 21 after cell transplantation. Immunofluorescence analysis for laminin (red) and lamin A/C (green). Hoechst was counterstained in blue. Scale bars: 25 µm. **(B)** Representative images of haematoxylin and eosin staining in transplanted muscles with microRNA-treated hMABs, non-treated hMABs, and cardiotoxin control at day 21 (d21). Scale bars: 100 µm. **(C)** Quantification of fiber size (mean cross-sectional area (CSA)) at day 21. **(D)** Quantitative frequency distribution analysis of the cross-sectional area of fibers at day 21. **(E)** Representative images of haematoxylin and eosin staining in transplanted muscles with microRNA-treated hMABs, non-treated hMABs, and cardiotoxin control at day 35 (d35). Scale bars: 100 µm. **(G)** Quantification of fiber size (mean CSA) at day 35. **(F)** Percentage of centronucleated fibers at day 35. **(H, I)** Masson’s tricrome staining of injected hindlimb muscles at day 21 (d21) and quantification of area of fibrosis. One-way ANOVA was used, and results are displayed as mean ± s.e.m (*p < 0.05, n = 4-5 muscles/group).

Next, we wanted to elucidate whether our selected miRNAs were able to enhance the myogenic contribution of hMABs *in vivo*. Recent evidence has shown that hMABs derived from young donors engraft and regenerate skeletal muscle when injected into dystrophic mice (11). Additionally, the enhanced myogenic performance of hMABs from young donors, with respect to hMABs from elderly donors can be assessed in the presence of an acute injury. Thus, in order to see whether the identified miRNAs are able to increase muscle hypertrophy when administered *in vivo*, we injected miRNA-treated and non-treated MABs intramuscularly in immunodeficient aged mice (≥ 18 months old) after an acute muscle injury induced by cardiotoxin (**Figures 4**, **5**). In order to monitor the biodistribution and the survival of the injected MABs, we established MABs stably expressing eGFP and luciferase (Luc) reporter proteins by retroviral vector transduction. GFP+ Luc+-transduced MABs were still capable of differentiating into fully-formed chimeric myotubes (**Figure 4A**). Forty-eight hours before injections, GFP+ Luc+ MABs were treated with miR-1 and miR-208a mimics, and cardiotoxin damage was induced in the hindlimb muscles of aged mice. Forty-eight hours later, miRNA-treated MABs and non-treated control MABs were injected intramuscularly into the hindlimb muscles (TA, GM, and Q) of aged mice. The distribution and survival of treated MABs were monitored by bioluminescence up to day 21 (**Figure 4B**). We did not detect any significant differences in the survival of treated and non-treated MABs at this time point; however, we observed a peak of the signal in both conditions at day 3 (**Figure 4B**). At day 21 (and furthermore at day 35), aged mice were euthanized and muscles were dissected. We noticed a significant increase in the weight of the hindlimb muscles injected with miRNA-treated MABs compared to the non-treated MABs, as well as controls (**Figure 4C**). Functionally, we observed that EDL muscles of the limbs injected with miRNA-treated MABs showed a stronger tetanic force trend (**Figure 4D**). MAB localization and presence in the injected muscles were visualized by immunofluorescence analysis for hLMNA and laminin. Both miRNA-treated and non-treated MABs were mainly detected in the interstitial space among fibers (**Figure 5A**). Interestingly, morphometric analysis of the injected muscles revealed that injected TA muscles with treated MABs showed a higher frequency of fibers with a larger cross-sectional area in comparison to TA muscles injected with non-treated MABs, as well as an overall increase in fiber cross-sectional area (CSA), at day 21 (**Figures 5B–D**).

Furthermore, at day 35, muscles injected with treated MABs and non-treated MABs displayed a similar percentage of centronucleated fibers, comparable to controls (**Figures 5E, F**). However, treated MABs contributed to a significant increase in the cross-sectional area of the TA compared to both non-treated MABs and controls (**Figure 5G**).

Finally, fibrosis, as shown by Masson’s staining, was reduced only in muscles injected with miRNA-treated MABs (**Figures 5H, I**).

These results showed that the myogenic contribution of hMABs *in vivo* after acute muscle injury was enhanced upon treatment with miR-1 and miR-208a.

## 4 Discussion

The balance between pathways inducing hypertrophy and atrophy in striated muscle is a crucial aspect to consider when approaching therapeutic strategies for skeletal muscle wasting (4). In this work, we have used the information obtained from animal models displaying either muscle hypertrophy or muscle wasting, in the latter case in both disease- and age-associated conditions. The rationale behind our analysis was to identify common dysregulated signatures with the final goal of identifying players that could enhance muscle regeneration. Given the growing evidence implicating circulating EVs as paracrine mediators in tissue regeneration (41), we screened the content of EVs derived from murine plasma. We obtained strong evidence that these EVs modulate the myogenic differentiation of both C2C12s as well as hMABs. It has been suggested that most circulating EVs might come from the skeletal muscle since it is one of the largest organs in the human body (42). Recently, Mytidou et al. confirmed that exosomes carrying the myomiRs could be found circulating in the serum of mice (43). Similarly, our results show that the miRNA content loaded into the circulating EVs of murine models of muscular hypertrophy correlates to the expression of those miRNAs in hypertrophic skeletal muscles (**Supplementary Figure 3**). Our analysis of the EV cargo has encompassed both RNA species (mRNAs and miRNAs) and proteins, resulting in the identification of two main miRNAs, miR-1 and miR-208a, which may impact the muscle phenotype.

miRNAs are largely implicated in myogenesis and muscle regeneration, with evidence reporting that miRNAs can be detected in EVs (44). It has been reported that the miRNA content of muscle-derived EVs is significantly altered with age (45). It is therefore not surprising that in our system EV-derived miRNAs appear to drive the beneficial effect on myogenic progenitors. miR-1 is a known modulator of hypertrophy, both in skeletal and cardiac muscles, where its overexpression is beneficial for reducing cardiac hypertrophy (46, 47). Supporting this evidence, we have identified target miRNAs in two different models of skeletal muscle hypertrophy (*Mstn-null* and Magic-F1 mice), where cardiac hypertrophy has a mild phenotype. Indeed, *Mstn-null* mice do not display any signs of cardiac hypertrophy, while Magic-F1 animals have an attenuated cardiac phenotype (40). It has also been shown that the overexpression of miR-1 inhibits cardiac fibroblast proliferation, alluding to its potential role in immune regulation as well (48). Similarly, miR-208a was among the most abundant circulating EV-derived miRNAs detected in both models. Albeit considered a specific cardiac miRNA, miR-208a is also a known regulator of *myostatin*, together with miR-208b and miR-499 (49, 50), implying a potential role in driving skeletal muscle hypertrophy that thus far remained unexplored. This work proposes miR-208a as an additional modulator of myogenic commitment of progenitor cells. The identification of the same EV-derived signature in two diverse murine models of skeletal muscle hypertrophy is compelling and further suggests that these miRNAs are crucial players in maintaining the phenotype. Although the latter statement is still speculative, we provided evidence that combined miR-1 and miR-208a treatments represent a potent option to increase the myogenic commitment of hMABs. However, further experiments need to be designed to address in detail, questions regarding the mechanism of these miRNAs and identifying their specific targets. However, we can hypothesize that both miRNAs regulate common pathways based on several shared predicted targets, such as members of the transforming growth factor β (TGFβ) pathway (TGFBR2, TAB2), SMAD4/6, and common epigenetic modulators (histone deacetylase 3 (HDAC3)). Despite our broad analysis of the EV cargo, it must be noted that we have not investigated other RNA species that have been implicated in EV-mediated modes of action and intercellular communication such as long non-coding RNAs (51).

When screening the mRNA content of EVs, we did not detect any differentially expressed genes, and we, therefore, concluded that mRNAs are stochastically entrapped in EVs in our setting, not likely being responsible for the enhanced myogenic effect. Many causes can be hypothesized, among them, the low number of transcripts detected. This data is consistent with other accounts reporting deep sequencing of EV-derived mRNAs (52, 53). Despite our analysis being statistically robust, increasing the number of samples might be beneficial to unravelling mRNAs possibly involved in the observed phenotype. Our mass spectrometry analysis, although preliminary, resulted in three important nbsp; observations: first, there is a high number of immunoglobulins present in all preparations; second, all preparations share expression of CD5-antigen like, which has been described as a novel marker for plasma-derived EVs (54). Lastly, we detected a significant increase of integrin alpha 2b in dystrophic and, to a lesser extent, in hypertrophic samples. While integrin alpha 2b is not highly expressed in normal muscle, it has been found to be upregulated upon cardiotoxin injury in mice (55). The source of this phenomenon was found to be activated satellite cells. Given the upregulation of integrin alpha 2b in dystrophic EV samples, the significance may imply a mechanism for satellite cell differentiation mediation in the constantly regenerative state of muscle, which is an apparent characteristic of muscular dystrophy. Analysis of additional samples will provide more insight into the protein content and allow a comprehensive understanding of the EV cargo.

The myogenic effect of the identified miRNA signature *in vitro* on hMABs was striking, and we observed a strong enhancement in myogenic differentiation. When transplanting miRNA-treated MABs *in vivo* in aged mice after acute muscle injury, we had less discriminative yet potent results. Muscle functionality was not significantly improved, although we observed a trend in strength improvement, and we detected hypertrophic remodelling as shown by an increase in muscle weight increase in muscle weight and fiber size. Our results also imply that miRNA treatment to MABs prior to intramuscular injection leads to a greater cross-sectional area of the TA muscle compared to both non-treated MABs injection as well as controls, without a correlation to the number of centronucleated fibers at day 35. This could mean that treated MABs contribute to hypertrophic fibers without affecting the rate of regeneration. We also showed a significant reduction in fibrosis when comparing muscles injected with treated MABs to non-treated MABs. Fibrosis is associated with poor recovery after injury in aged individuals (56, 57). We acknowledge that additional strategies can be implemented to refine our approach, for instance, stronger modulation of miRNAs by creating a stable cell line. Furthermore, given our *in vitro* results, miR-1 and miR-208a might directly affect and modulate resident myogenic stem cells. Novel methods to activate stem cells *in situ* are particularly demanding if moving towards a cell-free method of tissue-specific regeneration is desired. In this scenario, EVs hold an ideal advantage, since they are less immunogenic than stem cells, readily deliverable, and can be designed to carry tailored messages to target cells. Recent evidence has shown that EVs loaded with a specific cargo can be successfully delivered to skeletal muscle by adding modified, dystrophin splice-correcting phosphorodiamidate morpholino oligomers (EXOPMO) (58). In light of this result, a follow-up of our work would comprise the production of engineered EVs that could deliver miR-1 and miR-208a directly to target myogenic cells, generating a cell-free approach to target muscle wasting.

## Data availability statement

The proteomics data presented in the study are deposited in the ProteomeXchange Consortium via the PRIDE partner repository, accession number PXD035446 (doi: 10.6019/PXD035446). The RNA-sequencing data presented in the study are deposited in the GEO repository, accession number GSE216414.

## Ethics statement

The animal study was reviewed and approved by Ethical Approval of KU Leuven (P202/2016, P175/2016 with expansion).

## Author contributions

GG and EM-S participated in conception and design, collection and assembly of data, data analysis, interpretation, and manuscript writing with the help of LY, LDW, GMB, BKvdV, AR, and MS. LY and NG conducted the experiments for the revision. RD participated in proteomics experiments, data analysis and interpretation, and reviewed the manuscript. ÁC-C participated in the handling and analysis of the RNA sequencing data KPK, SQ, MQ, HG, and RG participated in data analysis and interpretation and reviewed the manuscript. SS, MB, and EF aided with the TEM imaging. All the authors gave input on data interpretation, reviewed the manuscript, and gave final approval for manuscript submission.

## Funding

The work done in the authors’ laboratory is supported by The Research Foundation Flanders (FWO) (#G066821N), INTERREG – Euregio Meuse-Rhine (GYM,Generate your muscle 2020-EMR116), and the Italian Ministry of Health, Ricerca Finalizzata (RF-2019-12369703). LY was supported by KU Leuven Rondoufonds voor Duchenne Onderzoek (EQQ-FODUCH-O2010) and MS is a recipient of the Hercules Foundation grant (AKUL/19/34).

## Acknowledgments

The authors gratefully acknowledge Sylvia Sauvage for her technical assistance, as well as Christina Vochten and Vicky Raets for their administrative assistance. The authors would also like to thank Louise Maeder and Vittoria Marini for their helpful contributions.

## Conflict of interest

The authors declare that the research was conducted in the absence of any commercial or financial relationships that could be construed as a potential conflict of interest. The Associate Editor YT and the reviewer CS co-authored publications with the corresponding author MS. MS declared that his connection to YT is limited to professional acquaintance with no shared grants, supervisory ties, or direct collaborations beyond YT’s historical contribution of biological samples to MS’s former supervisor’s group, and his connection to CS is restricted to co-authoring a single publication in 2008 in which neither served as a senior or corresponding author, with no subsequent scientific or financial collaborations. The authors declare that the identity of the reviewers was not revealed to them during the peer-review process.

An investigation by the Research Integrity team found that this conflict of interest did not unduly impact the peer review process or scientific validity of the article.

## Correction note

A correction has been made to this article. Details can be found at: 10.3389/fimmu.2026.1945625.

## Publisher’s note

All claims expressed in this article are solely those of the authors and do not necessarily represent those of their affiliated organizations, or those of the publisher, the editors and the reviewers. Any product that may be evaluated in this article, or claim that may be made by its manufacturer, is not guaranteed or endorsed by the publisher.

## Supplementary material

Supplementary Figure 1 RNA-sequencing and proteomic analysis of extracellular vesicles. **(A)** Transcripts content in extracellular vesicles (EVs) from different conditions (Wild type (WT), dystrophic (Dys), and hypertrophic (Hyp)) used for RNA-sequencing (RNA-seq). **(B)** RNA-seq principal component analysis plot shows a stochastic distribution of the different samples. **(C)** Venn diagram of shared genes in the different conditions. WT and Dys EVs share a higher number of genes. **(D)** Heatmap representing the 23 shared genes among all conditions identified in the RNA-seq analysis. **(E)** Venn diagram of shared proteins among all conditions. **(F)** Heatmap displaying proteins (obtained after data analysis with Progenesis relative quantification software) that are discriminating between the different conditions. Results are subjected to hierarchical clustering and applying a Kruskal-Wallis non-parametric analysis on the total list of identified proteins (using Scaffold software), only integrin alpha 2b and antithrombin-III showed a significant (p < 0.05) difference among the conditions.

Supplementary Figure 2 Modulation of some of the top differentially expressed microRNAs did not lead to any myogenic differentiation improvement. **(A)** Heatmap of selected microRNAs (miRNAs) expressed in hypertrophic (Hyp)-, dystrophic (Dys)-, and aged-derived extracellular vesicles (EVs), analyzed by qPCR analysis using the mmu-miRNome microRNA profiling kit. **(B)** Detailed expression levels of some of the highest differentially expressed miRNAs in the EVs from the different conditions (miR-150*, miR-574-5p, and miR-30e*). **(C, D)** Modulation of miR-150*, miR-574-5p, and miR-30e* using miRNA mimics or antagomirs did not show a significant enhancement of myogenic differentiation in human mesoangioblasts, as shown by immunofluorescence staining **(B)** for myosin heavy chain (MyHC) (red) and lamin A/C (green) and fusion index quantification **(C)**. For c, scale bars: 100 µm. For d, a two-tailed Student’s t-test was used, and results are displayed as mean ± s.e.m (n=3, *p<0.05).

Supplementary Figure 3 microRNA content in the hindlimb muscles of mice. **(A)** Selected microRNA (miRNA) content (miR-1, miR-133a, miR-206, and miR-208a) in the hindlimb skeletal muscles of wild type (WT), hypertrophic (Hyp), dystrophic (Dys), and aged mice. **(A)** Analysis of the selected miRNAs in extracellular vesicles derived from Magic-F1 mice compared to WT mice. One-way ANOVA **(A)** and two-tailed Student’s t-test **(B)** were used, and results are displayed as mean ± s.e.m (n=3, *p<0.05).
