# Correction: Extracellular vesicle-derived miRNAs improve stem cell-based therapeutic approaches in muscle wasting conditions

**DOI:** 10.3389/fimmu.2026.1945625

**Published:** 2026-09-10

**Authors:** Frontiers Editorial Office

**Affiliations:** Frontiers Media SA, Lausanne, Switzerland

**Keywords:** extracellular vesicle, hypertrophy, muscular dystrophy, aging, miRNA, skeletal muscle

Due to an oversight during review, an existing **Conflict of interest** (COI) between the “Topic Editor, Yvan Torrente (YT), and one of the reviewers, Clara Sciorati (CS), and the Corresponding author, Maurilio Sampaolesi (MS)”, was not declared. The updated **Conflict of interest** statement can be seen below. The undeclared COI did not impact on the quality of the peer review reports. Additionally, our review of the raw data provided by the authors confirmed there are no research integrity concerns.

The publisher apologizes for this mistake.

The original article has been updated.

The **Conflict of interest** statement was erroneously given as “The authors declare that the research was conducted in the absence of any commercial or financial relationships that could be construed as a potential conflict of interest.”

The correct **Conflict of interest** statement is “The authors declare that the research was conducted in the absence of any commercial or financial relationships that could be construed as a potential conflict of interest. The Associate Editor YT and the reviewer CS co-authored publications with the corresponding author MS. MS declared that his connection to YT is limited to professional acquaintance with no shared grants, supervisory ties, or direct collaborations beyond YT’s historical contribution of biological samples to MS’s former supervisor’s group, and his connection to CS is restricted to co-authoring a single publication in 2008 in which neither served as a senior or corresponding author, with no subsequent scientific or financial collaborations. The authors declare that the identity of the reviewers was not revealed to them during the peer-review process.

An investigation by the Research Integrity team found that this conflict of interest did not unduly impact the peer review process or scientific validity of the article.”

