# Supplementary figures and images for: Extracellular vesicle-derived miRNAs improve stem cell-based therapeutic approaches in muscle wasting conditions
Source: Front Immunol. 2022 Nov 14;13:977617. doi: 10.3389/fimmu.2022.977617 (PMC9702803; doi:10.3389/fimmu.2022.977617)

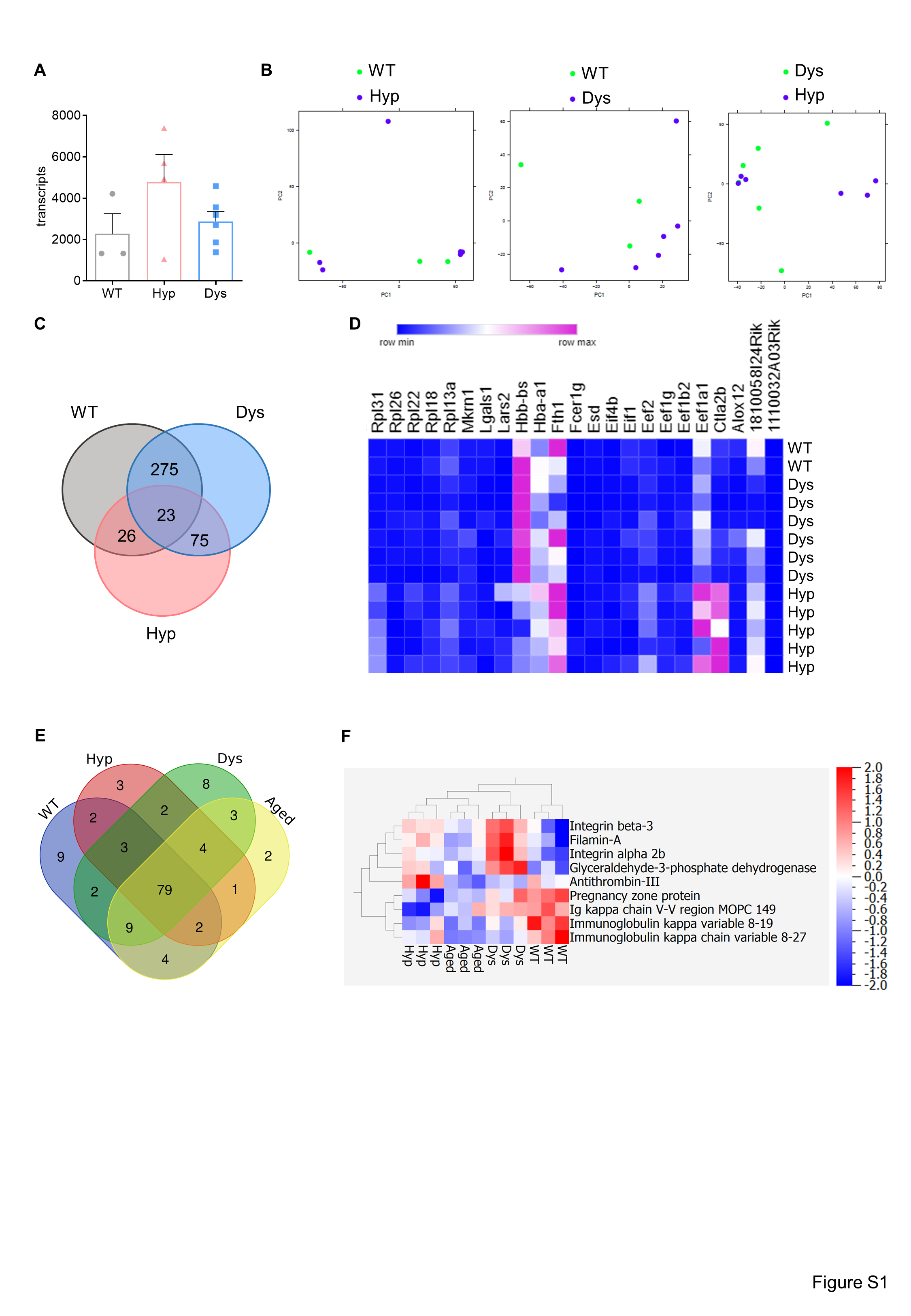

Supplement: Supplementary Figure 1 — RNA-sequencing and proteomic analysis of extracellular vesicles. (A) Transcripts content in extracellular vesicles (EVs) from different conditions (Wild type (WT), dystrophic (Dys), and hypertrophic (Hyp)) used for RNA-sequencing (RNA-seq). (B) RNA-seq principal component analysis plot shows a stochastic distribution of the different samples. (C) Venn diagram of shared genes in the different conditions. WT and Dys EVs share a higher number of genes. (D) Heatmap representing the 23 shared genes among all conditions identified in the RNA-seq analysis. (E) Venn diagram of shared proteins among all conditions. (F) Heatmap displaying proteins (obtained after data analysis with Progenesis relative quantification software) that are discriminating between the different conditions. Results are subjected to hierarchical clustering and applying a Kruskal-Wallis non-parametric analysis on the total list of identified proteins (using Scaffold software), only integrin alpha 2b and antithrombin-III showed a significant (p < 0.05) difference among the conditions. [file Image_1.tif]

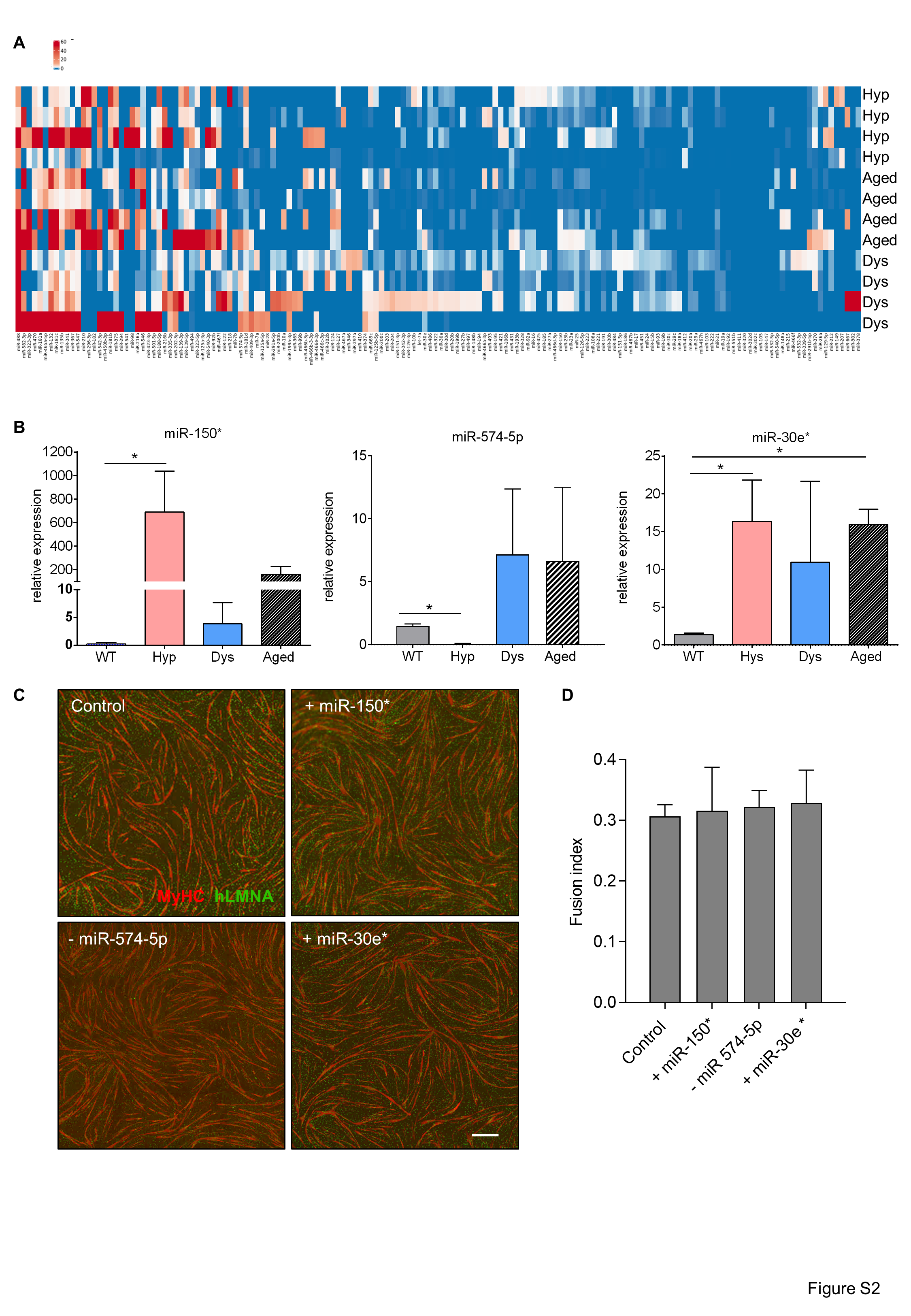

Supplement: Supplementary Figure 2 — Modulation of some of the top differentially expressed microRNAs did not lead to any myogenic differentiation improvement. (A) Heatmap of selected microRNAs (miRNAs) expressed in hypertrophic (Hyp)-, dystrophic (Dys)-, and aged-derived extracellular vesicles (EVs), analyzed by qPCR analysis using the mmu-miRNome microRNA profiling kit. (B) Detailed expression levels of some of the highest differentially expressed miRNAs in the EVs from the different conditions (miR-150*, miR-574-5p, and miR-30e*). (C, D) Modulation of miR-150*, miR-574-5p, and miR-30e* using miRNA mimics or antagomirs did not show a significant enhancement of myogenic differentiation in human mesoangioblasts, as shown by immunofluorescence staining (B) for myosin heavy chain (MyHC) (red) and lamin A/C (green) and fusion index quantification (C). For c, scale bars: 100 µm. For d, a two-tailed Student’s t-test was used, and results are displayed as mean ± s.e.m (n=3, *p<0.05). [file Image_2.tif]

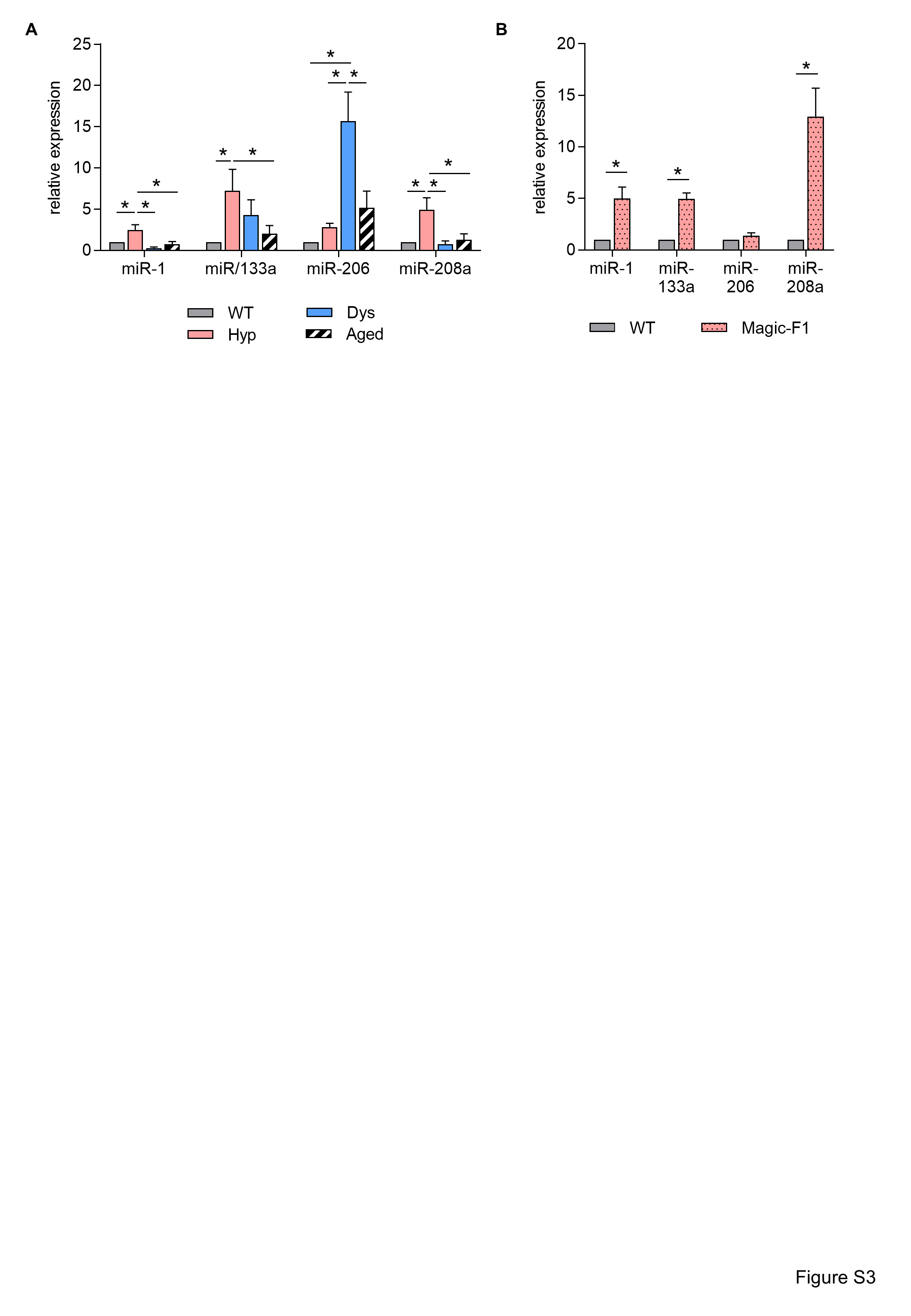

Supplement: Supplementary Figure 3 — microRNA content in the hindlimb muscles of mice. (A) Selected microRNA (miRNA) content (miR-1, miR-133a, miR-206, and miR-208a) in the hindlimb skeletal muscles of wild type (WT), hypertrophic (Hyp), dystrophic (Dys), and aged mice. (A) Analysis of the selected miRNAs in extracellular vesicles derived from Magic-F1 mice compared to WT mice. One-way ANOVA (A) and two-tailed Student’s t-test (B) were used, and results are displayed as mean ± s.e.m (n=3, *p<0.05). [file Image_3.tif]
